# Comparison of quantitative imaging analysis methods to evaluate murine [18F]FLT PET therapy response studies

**DOI:** 10.1186/s12967-025-07475-2

**Published:** 2025-11-26

**Authors:** J. D. Kalen, J. L. Tatum, P. M. Jacobs, J. H. Doroshow

**Affiliations:** 1 Small Animal Imaging Program, Frederick National Laboratory for Cancer Research, Frederick, MD USA; 2 Office of the Director, Division of Cancer Treatment and Diagnosis, National Cancer Institute NIH, Bethesda, MD USA

**Keywords:** FLT, PET, Preclinical imaging, Diagnostic, Therapy response

## Abstract

**Background:**

Positron Emission Tomography (PET) imaging using 3’-deoxy-3’-[F-18] fluorothymidine (FLT) is a valuable non-invasive marker of tumor proliferation. However, variability in imaging protocols and quantitative analysis techniques hampers comparability across preclinical chemotherapy response studies.

**Methods:**

To address this issue in preclinical studies, we evaluated two key areas: (1) imaging standardization (2) semi-quantitative analyses, including Standard Uptake Value (SUV) SUV(max) and SUV(mean), and percent injected (%ID)/g. Using standardized methods of animal handling, image acquisition, image reconstruction and analyses, we assessed multiple FLT uptake metrics in three responsive Patient-Derived Xenograft (PDX) models treated with temozolomide/berzosertib or cisplatin/berzosertib. Metrics included: %ID, SUVbw(max), SUVbw(mean) with a Region of Interest (ROI) threshold of 50% of the maximum value [SUVbw(mean-50%)], and the ratios tumor-to-tissue and tumor-to-liver. Statistical comparisons were made using Brown-Forsythe and Kruskal-Wallis ANOVA and unpaired two tailed t-test to assess cohort differences.

**Results:**

Across models and timepoints, no statistical difference among analysis techniques were observed except for one outlier at day 3 in one vehicle cohort, likely due to variability in the normalization parameter. SUVbw(max) and SUVbw(mean-50%) showed the strongest ability to differentiate between vehicle and treated cohorts.

**Conclusion:**

Semi-quantitative FLT PET metrics can be harmonized across preclinical studies, with caveats in understanding the normalization parameters, with SUV-based metrics preferred and that larger numbers of animals per cohort will be required to power (≥0.8) robust conclusions. These findings support FLT PET standardization in translational imaging.

**Supplementary Information:**

The online version contains supplementary material available at 10.1186/s12967-025-07475-2.

## Background

Oncologists use an array of assays, ranging from in vivo imaging to liquid biopsies, RNA-Seq, serum, and tissue biopsies (H&E staining), to assess the efficacy of therapeutic modalities. This array of technologies offers critical measurements to assist the oncologist in treating a patient [1, 2], by identifying the appropriateness of therapies and evaluating response to therapy [3].

PET imaging with FLT is considered a quantitative marker of proliferation, a cancer hallmark, that measures S-phase phosphorylation by thymidine kinase 1. However, non-clinical studies of FLT as a response marker for combination therapy treatments have been inconsistent in animal handling, image acquisition, and image analysis making it difficult to aggregate results among the numerous small studies in pre-clinical publications [4, 5].

In vivo tracer imaging techniques using serial imaging to evaluate a drug’s effect on molecular pathways require robust analysis methods to be useful and comparable across studies, particularly when evaluating preclinical studies. The robustness of SUV(peak) [6] has been demonstrated in clinical studies, whereas others have demonstrated high variability in SUV(max) and SUV(mean) [7] for tumors < 5 mL (diameter < 2.1 cm) in human patients. Unfortunately, the SUV(peak) used in clinical imaging employs a standard region of interest (ROI), approximately 1.2 cm diameter around the maximum peak value that is difficult to apply in preclinical studies. Murine PET studies enroll animals when tumors are approximately 0.5 cm or less in diameter and typically assess drug responses over several weeks with rapidly growing tumors, so the large ROI required for SUV(peak) is not practical to implement. Preclinical researchers have also used other analytical approaches for their computational simplicity, for example tumor uptake normalized to injected dose (%ID) or comparison of the tumor activity within a standard ROI to an organ (liver or muscle) for evaluating drug efficacy.

Validating FLT as a valuable in vivo biomarker for precision oncology in the preclinical arena requires two steps that we address in this report:

1. Imaging Standardization: In the reported literature there is little standardization in animal handling, imaging acquisition, and imaging data analysis, leading to data inconsistencies across studies. Various imaging medical societies and researchers have addressed some of these issues [8–13]. For example, the uptake and pharmacokinetics of FLT may vary [14] due to the anesthetic (ketamine/xylazine, medetomidine/midazolam or isoflurane) employed. In addition to standardization of animal handling, acquisition, reconstruction algorithm and image analysis, guidance in reporting of these parameters is also required [15]. We have developed an extensive set of SOPs for pre-clinical imaging that are publicly available [Supplementary S1].
2. Semi-Quantitative Analyses: A review of preclinical and clinical publications reveals several methods that have been applied to FLT [SUV(max), SUV(mean), ΔSUV, K-Patlak, %ID/g, SUV(tumor)/SUV(tissue), SUV(tumor)/SUV(liver) and SUV(tumor)/SUV(blood)], mostly adapted from FDG although the uptake and retention mechanisms are dissimilar. Soloviev [16] performed a meta-analysis of several clinical FLT studies using different treatments and analysis techniques and concluded that there is need for a systematic comparison due to the variety of clinical endpoints and analysis techniques. We therefore compared the response to treatment using several common methods in three clinically responsive Patient-Derived Xenograft (PDX) treatment models.

## Materials and methods

### Mouse models

Animal studies were performed according to the Frederick National Laboratory for Cancer Research (Frederick, MD) IACUC guidelines (IACUC Protocols No. 16–008, mods 8, 10, and 26). Tumor fragments (2x2x2 mm^3^) ([urothelial/bladder cancer, NOS: BL0293–F563], [transitional cell carcinoma/urothelial: BL0479–F1894] and [squamous cell lung carcinoma: 765638-272-R]), obtained from the National Cancer Institute Patient-Derived Models Repository (PDMR) [17] were implanted [SOP in Supplementary S2] into the right flank of appropriate gender NOD-*scid* gamma (NSG) mice (The Jackson Laboratory, Bar Harbor, ME). The mouse gender matched the gender of the patient from whom the tissue was acquired. Table 1 provides the details for each PDX study: fragment code, the National Cancer Institute Cancer Therapy Program (CTEP) Simplified Disease Classification (SDC) [18], and gender.

**Table 1:** Patient-derived xenograft (PDX) models with corresponding disease classification (CTEP) and donor gender

PDMR Fragment	CTEP SDC designation	Gender
BL0293–F563	Urothelial/bladder cancer, NOS	Female
BL0479–F1894	Transitional cell carcinoma/urothelial	Male
765638-272-R	Squamous cell lung carcinoma	Female

### Drug challenge studies

**Fig. 1:**
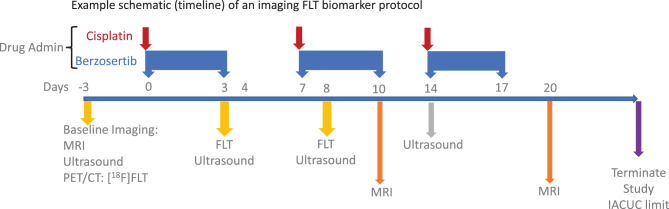
Simplified schematic (timeline) of an example imaging FLT biomarker protocol: PDX model 765638-272-R (squamous cell lung carcinoma). Drug dosage and route: cisplatin (3 mg/kg; ip; Q7Dx2) and berzosertib (60 mg/kg; po; QDx4-3 cycles). Timeline (days) are with respect to the first day of drug administration (day 0)

The xenograft growth was measured weekly via caliper measurements (*V* (mm^3^) = {π/6 ×(Length ×Width ×Height)}, and mice were enrolled into the study when the xenografts reached the enrollment criteria (200 ± 25 mm^3^). Within each study, a single investigator performed the caliper measurements. Each cohort (vehicle, single and combination) contained 6 mice except for the squamous cell lung carcinoma (765638-272-R) drug challenge which enrolled 5 mice per group (vehicle and cisplatin) and 7 mice in the combination group (cisplatin with berzosertib). Each drug study incorporated two drug cycles. The single drug arms are not included in this study of analysis methodologies. The details of the drug treatment and administration are shown in Table 2, and Figure 1 provides a simplified schematic (timeline) of an example imaging biomarker protocol.

**Table 2:** Drug treatment, number of mice per cohort, and administration route/schedule per PDX model-study. po: per os (oral), ip (intraperitoneal); QDx5: daily for 5 days, rest 2-days, repeat for 2–3 cycles; QDx4: daily for 4 days, rest-3 days, repeat 2–3 cycles; Q7Dx3: administer 7-day interval for 3 cycles. The single drug arm was not included in this study of analytic methods. Temozolomide is a dna alkylating agent; berzosertib inhibits the enzyme ataxia telangiectasia and Rad3 related (atr); cisplatin is a dna cross-linking agent

PDMR fragment	Drug treatment	# of mice in cohort	Drug route	Schedule
BL0293–F563	Vehicle	6	PO	QDx5
	Temozolomide (50 mg/kg)	6	PO	QDx5
	Temozolomide (50 mg/kg) & Berzosertib (45 mg/kg)	6	PO	QDx5 QDx4
BL0479–F1894	Vehicle	6	PO	QDx5
	Temozolomide (50 mg/kg)	6	PO	QDx5
	Temozolomide (50 mg/kg) & Berzosertib (45 mg/kg)	6	PO	QDx5 QDx4
765638-272-R	Vehicles	5	IP PO	Q7Dx3 QDx4
	Cisplatin (3 mg/kg)	5	IP	Q7Dx3
	Cisplatin (3 mg/kg) & Berzosertib (60 mg/kg)	7	IP PO	Q7Dx3 QDx4

### Imaging

Advanced imaging was initiated when tumors reached (200 ± 25 mm^3^) and consisted of FLT positron emission tomography/x-ray computed tomography (PET/CT) (Inveon Multi-Modality PET/CT, Siemens Medical Solutions, Knoxville, TN) and ultrasound (Vevo2100, FujiFilm/VisualSonics, Ontario, CA) performed on days 0, 3 or 4 and 8, and non-contrast T2w 3T MRI (Achieva, Philips Medical, Netherlands) at 7 or 10-day intervals until mice required euthanasia per IACUC guidelines (tumor dimension > 2 cm in any direction, or weight loss > 25%). The imaging protocols for PET/CT, MRI, ultrasound and animal preparation with physiological monitoring have been reported [19].

### PET/CT protocol

The average MBq activities (Mean ± SD) of FLT Intra-venous (IV) injected via tail-vein is shown in Table 3 for the three PDX models. After the injection, the mice (not fasted prior to the study) were then placed in a cage with water but no feed and remained awake during the uptake phase. Mice were placed under anesthesia (2% Isoflurane with oxygen as carrier at a flow of 1 L/min) and imaged in the prone position for a Step-and-Shoot CT for PET attenuation correction, followed by a 20-min PET acquisition initiated at approximately 1-h post injection. CT acquisition parameters were: 80 kVp, 500 μA, 200 ms per step, 120 steps covering 220°. PET list-mode data were acquired using an energy window of 350–650 keV and a 3.432 ns coincidence timing window. CT images were reconstructed using a cone beam algorithm resulting in 192 × 192 matrix and PET used Ordered Subset Expectation Maximization (OSEM-3D) with 12 subsets and 4 iterations resulting in a 256 × 256 matrix.

**Table 3:** FLT radiopharmaceutical IV-tail vein injection (MBq) (mean ± SD) per model/study

PDMR Fragment	FLT (MBq)
BL0293–F563	6.24 ± 0.66
BL0479–F1894	5.67 ± 0.78
765638-272-R	6.64 ± 0.71

Baseline imaging was performed on Day 0, with drug administration initiated after completion of PET imaging; on subsequent days drugs were administered early morning and PET commenced two-three hours after drug dosing. FLT PET/CT was performed in the first two drug cycles.

### PET image analysis

FLT PET/CT DICOM images were displayed and analyzed on a MIM workstation (v 6.6.5, MIM Software Inc, Cleveland, OH).

To compare the tumor radiopharmaceutical uptake between subjects (cohorts and animal models), a normalization technique must be implemented, such as SUV for the activity maximum, mean, or peak values normalized by the injected activity decayed to time of the scan and body weight or by using the subject as its own normalization with respect to an organ (such as liver, muscle, or other tissue), which does not require knowledge of the injected activity [20, 21], or the percent of total tumor activity with respect to the injected activity (%ID) decayed to the time of the scan.

FLT tumor uptake quantification used the following techniques: %ID, SUVbw(max), SUVbw(mean) with a threshold ROI of 50% of the maximum value [SUVbw(mean-50%)], and a ratio of the tumor to liver or muscle using the same 2D ROI. For easier comparison of the different analysis techniques for each PDX model, each value was normalized to its initial baseline, followed by calculations of averages and standard deviations for each cohort.

To evaluate differences between analysis techniques two ANOVA statistical tests were implemented, the Brown-Forsythe and Kruskal-Wallis (GraphPad Prism ver. 10.3.1). The Brown–Forsythe test is used when the samples distributions are of equal variances. The Kruskal–Wallis test is a non-parametric (non-normal distribution of the residuals) statistical test for evaluating whether samples originate from the same distribution, comparing two or more independent samples of equal or different sample sizes.

To evaluate differences between vehicle and treatment cohorts (therapeutic response evaluation) the unpaired two tailed t-test (GraphPad Prism ver. 10.3.1) was assessed for each quantiative method (vehicle vs treatment) at the end of the second drug cycle (Day 8) and also assessed for tumor volumes in the vehicle and combination treatment cohorts at the second drug cycle (Day 8) and after completing all drug administrations (Day 14 or Day 15).

Post-hoc power (1-β), effective size and the total number of samples required for a power of 0.8 were calculated with G*Power (ver. 3.1.9.7) [ref 32] based on the standard deviations in the present study.

## Results

**Fig. 2:**
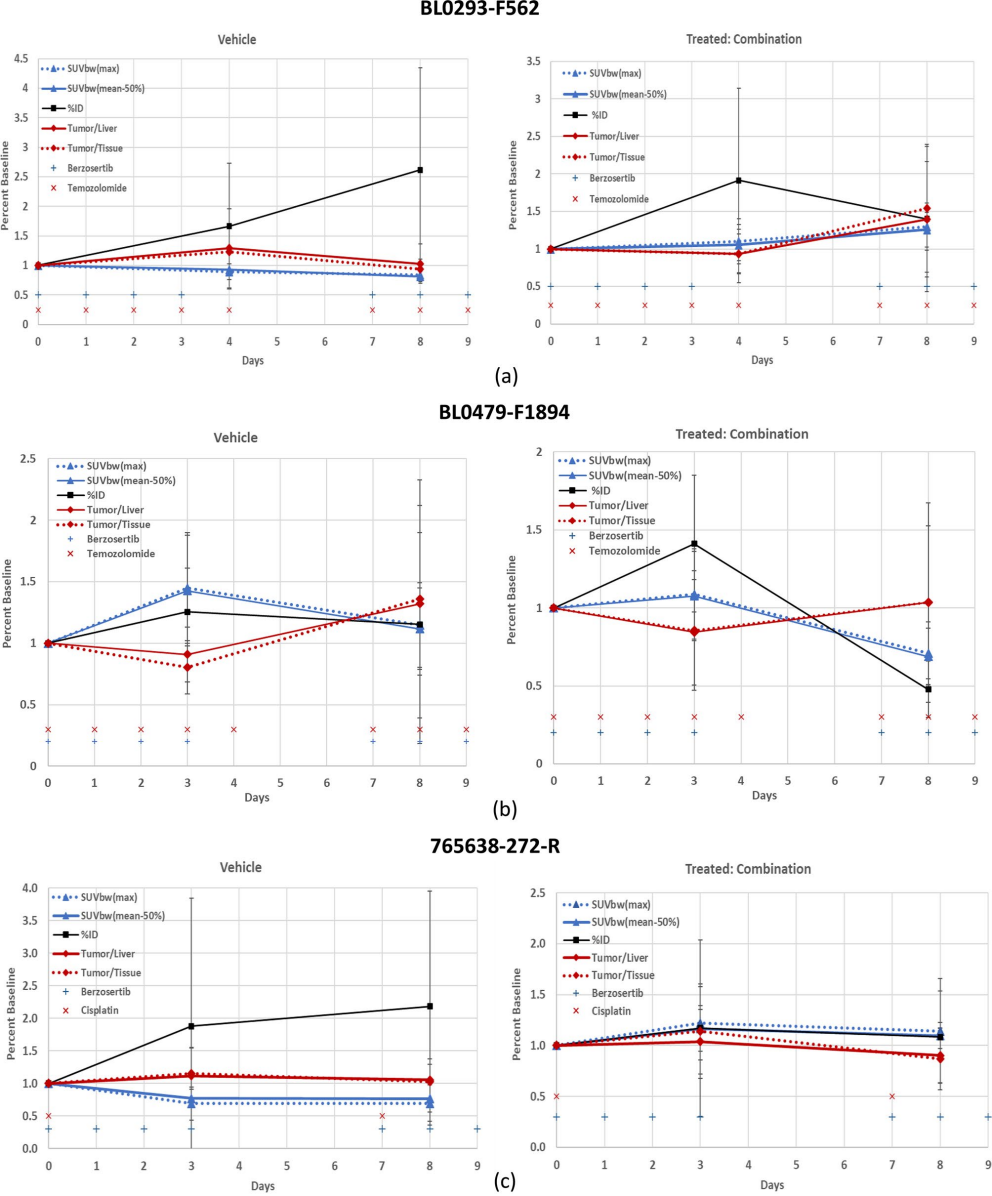
Quantitative methods normalized to baseline (mean ± sd) for (**a**) urothelial/bladder cancer, nos: BL0293–F563, (**b**) transitional cell carcinoma/urothelial cancer: BL0479–F1894 and (**c**) squamous cell lung carcinoma: 765,638-272-R. Vehicle (left panel) and combination drugs (right panel) treatment. Timeline (days) are with respect to the first day of drug administration (day 0) and the treatment administration timeline is also presented for reference. SUVbw [triangle] (max; dotted line and mean-50%; solid line); %id [square]; ratio [diamond] (Tumor/Liver; solid line and Tumor/Tissue; dotted line); treatment (berzosertib (+) and Cisplatin or Temozolomide (**x**))

Comparisons for each analysis technique with respect to baseline for each model are presented in Fig. 2; (a) (Urothelial/bladder cancer, NOS: BL0293–F563), (b) (Transitional cell carcinoma/urothelial cancer: BL0479–F194) and (c) (Squamous cell lung carcinoma: 765638-272-R). In addition to the comparisons of the techniques, the drug administration dosing schedule is provided; vehicle (left panel) and treatment combination drug cohorts (right panel) are also presented.

The ANOVA statistical analysis Brown-Forsythe and Kruskal-Wallis for comparing the analysis methods at days 3 or 4 and 8 for both vehicle and combination drug treatment for each PDX model is presented in Table 4. P-values < 0.05 are highlighted bold with asterisk.

**Table 4:** ANOVA: Brown-Forsythe and Kruskal-Wallis statistical analysis for comparing the methodologies within each cohort and time-point. P-values < 0.05 are highlighted bold with asterisk

		ANOVA p-value	
Model	Condition	Brown-Forsythe	Kruskal-Wallis
BL0293–F563	Vehicle-Day 4	0.325	0.400
	Vehicle-Day 8	0.070	0.164
	Combo-Day 4	0.177	0.199
	Combo-Day 8	0.971	0.989
BL0479–F1894	Vehicle-Day 3	0.013*	0.006*
	Vehicle-Day 8	0.959	0.542
	Combo-Day 3	0.064	0.066
	Combo-Day 8	0.113	0.033*
765638-272-R	Vehicle-Day 3	0.389	0.050
	Vehicle-Day 8	0.163	0.055
	Combo-Day 3	0.925	0.875
	Combo-Day 8	0.287	0.266

No statistical difference between analysis techniques (*p* < 0.05) within each cohort and time-point using the parametric Brown-Forsythe test or the Kruskal-Wallis non-parametric test were noted except for the PDX model BL0479–F1894 vehicle at day 3 for both statistical tests (*p* = 0.013 and 0.006, respectively) and the combination drug at day 8 for the Kruskal-Wallis method (*p* = 0.033), bold with an asterisk.

**Fig. 3:**
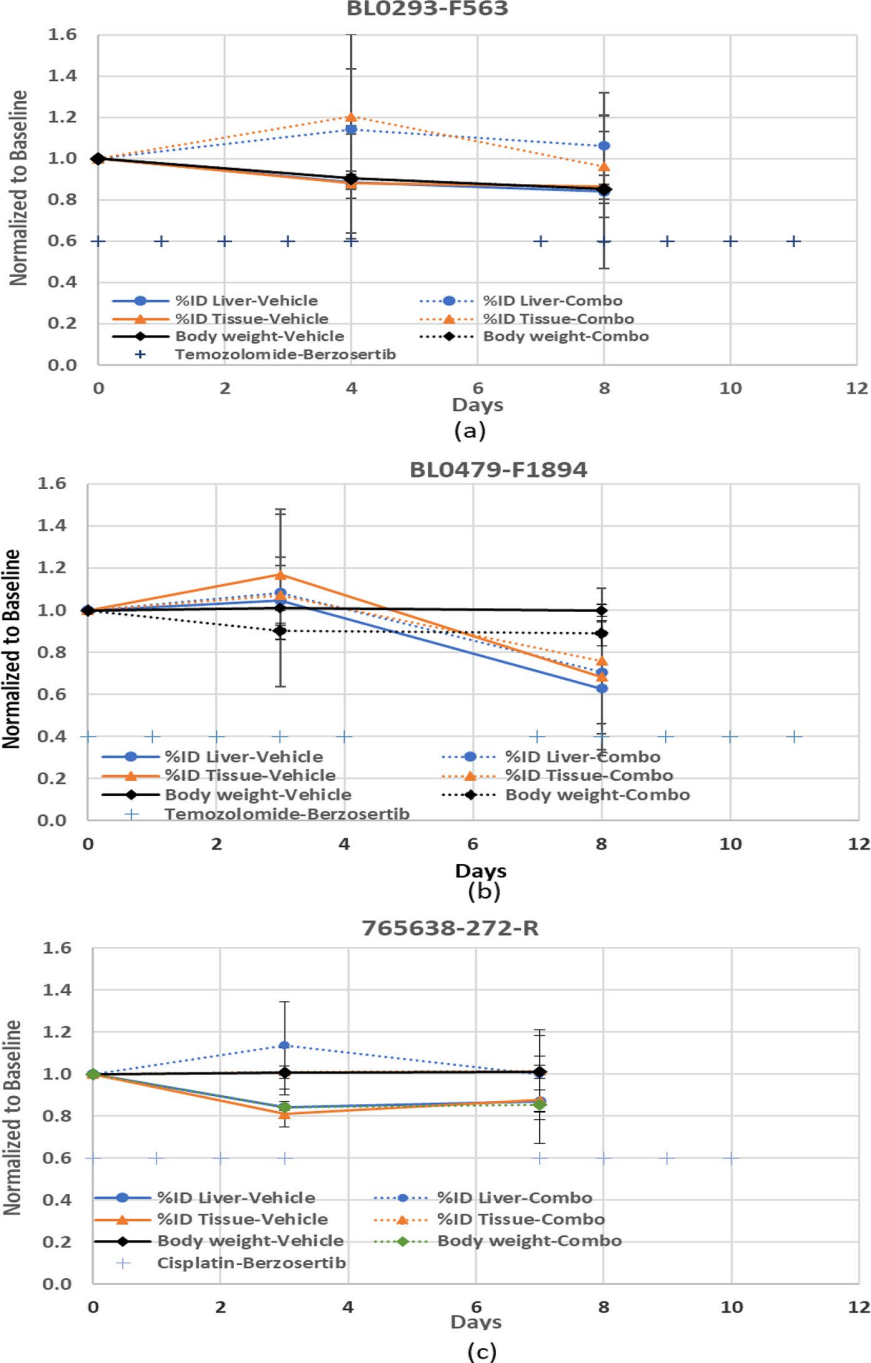
%id liver, %id Tissue; and body weights (mean ± sd) normalized to baseline for each of the models; (**a**) BL0293–F563 (urothelial/bladder cancer), (**b**) BL0479–F1894 (transitional cell carcinoma/urothelial cancer), and **c**) 765,638-272-R (squamous cell lung carcinoma). Treatment administration timeline [+] is also presented for reference. %id liver [circle], %id tissue [triangle], body weight [diamond]; (vehicle: solid line; treated-Combination; dotted line)

The low Kruskal-Wallis and Brown-Forsythe ANOVA p-values in the vehicle cohort in the BL0479–F1894 model at day 3, (*p* = 0.013 and *p* = 0.006, respectively) (Table 4) demonstrates that there is a statistical significance between some of the methodologies. A two-tailed Wilcoxon matched-pairs test revealed that the tumor/tissue ratio differed significantly from other methods (p-value = 0.03) with respect to SUV (max and mean) and %ID, while the other methods were not statistically significant. These statistical differences can be due to the change in the normalization parameters with respect to the baseline of the radiopharmaceutical uptake in the liver and tissue (%ID). Illustrated in Fig. 3, the %ID for the liver and tissue are not constant throughout the study, varying as much as 20–40% with respect to baseline, while body weights generally decline gradually throughout the study (−10 to −15%).

**Fig. 4:**
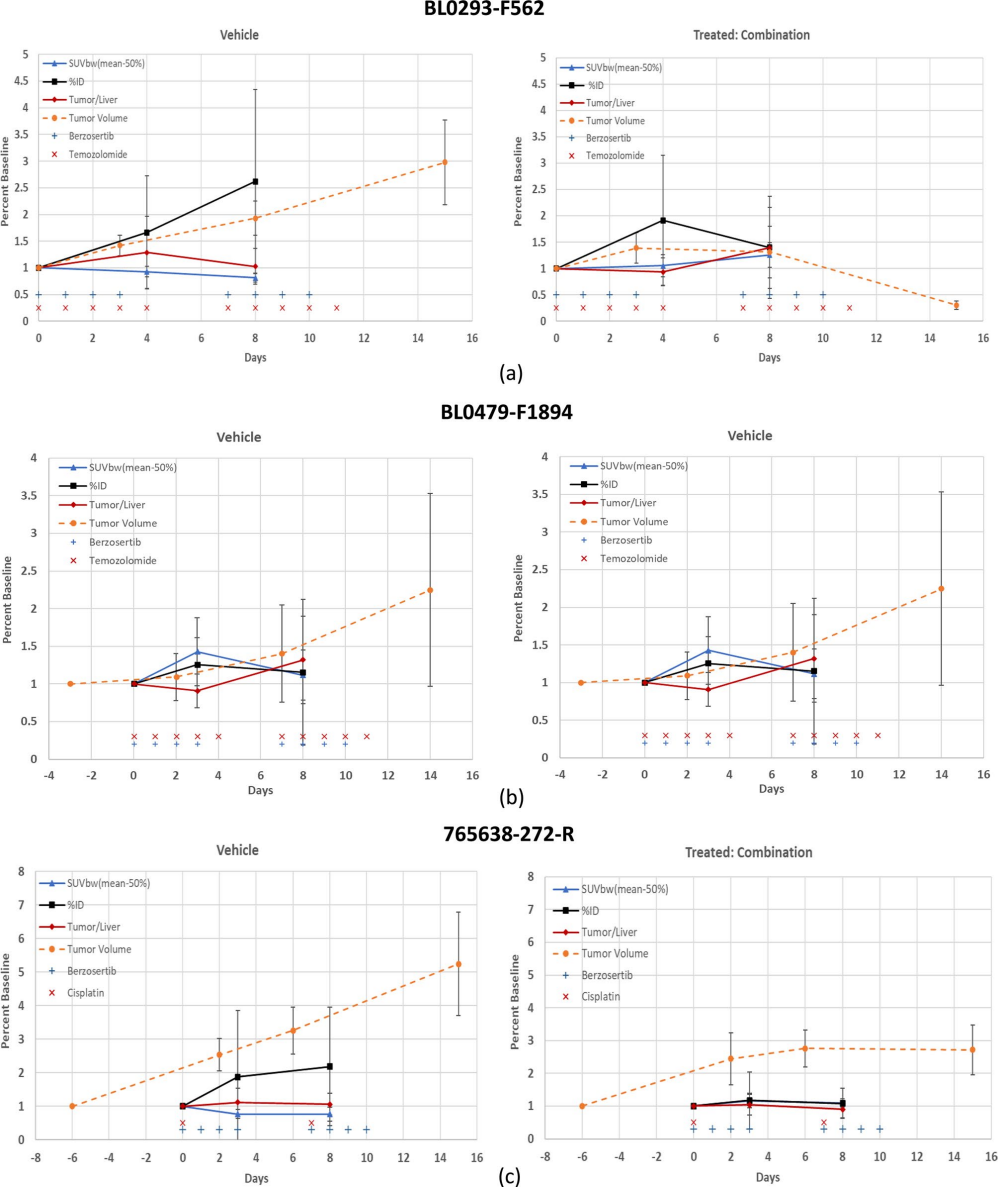
Subset of quantitative methods normalized to baseline (mean ± sd) and comparison to tumor volumes for (**a**) urothelial/bladder cancer, nos: BL0293–F563, (**b**) transitional cell carcinoma/urothelial cancer: BL0479–F1894 and (**c**) squamous cell lung carcinoma: 765638-272-R. Vehicle (left panel) and combination drugs (right panel) treatment. Timeline (days) are with respect to the first day of drug administration (day 0) and the treatment administration timeline is also presented for reference. SUVbw(mean-50%) [trangle]; %id [square]; tumor/Liver ratio [diamond]; tumor volume [circle-dashed line]; treatment (berzosertib (+) and Cisplatin or Temozolomide (x))

Statistically significant differences (*p* < 0.05 and power (1-β) > 0.8) (bold with asterisk) within a 95% confidence interval, shown in Table 5, along with power analysis (values in parenthesis), were observed between the vehicle and combination treatment groups for tumor volumes at day 14 (end of study) for the PDX model BL0293–F563 (*p* = 0.006; 1-β = 0.999). SUVbw (max) (*p* = 0.015; 1-β = 0.841) and SUVbw(mean-50%) (*p* = 0.004; 1-β = 0.971) for the PDX model BL0293–F563, were the only analysis techniques that also demonstrated statistical significance and high power between the vehicle and combination treatment cohorts at the end of the PET study, Day 8. The PDX model BL0479–F1894 demonstrated low *p* = 0.045 for comparison between vehicle and treated tumor volumes at day 14, and the quantitative methods SUVbw(max) and SUVbw(mean-50%) resulted with statistical significance with p-values (0.023 and 0.02), respectivley, but with low power (1-β = 0.651 and 0.683), respectively. No other analysis techniques demonstrated statistical significance between the vehicle and combination treatment. Figure 2 presented all of the quantitative methods and demonstrated that some of these methods are very similar and overlap: SUVbw (max) and SUVbw(mean-50%); and the ratios tumor-to-liver and tumor-to-muscle. To enhance the comparison of the quantitative methods to tumor volumes for comparison of the vehicle and treated cohorts (Fig. 4), one set of the similar quantitative methods, SUVbw(max) and the ratio tumor-to-muscle, were removed and the percent of baseline (y-axis) was retained, for easy comparison, for vehicle and treated cohorts of each PDX model.

**Table 5:** Vehicle vs treated-combination un-paired two-tailed t-test (95% confidence interval) and power (1-β) (parenthesis) for tumor volume and each analysis method at day 8 (end of pet study) and day 14 (end of study). P-values < 0.05 and power > 0.8 are highlighted bold with asterisk

	PDX Model		
Analysis Technique	BL0293–F563	BL0479–F1894	765,638-272-R
Tumor Volume (Day 14)	0.006* (0.999)*	0.045* (0.650)	0.051 (0.880)*
Tumor Volume (Day 8)	0.448 (0.627)	0.960 (0.078)	0.128 (0.222)
SUVbw(max) (Day 8)	0.015* (0.841)*	0.023* (0.651)	0.142 (0.348)
SUVbw(mean-50%) (Day 8)	0.004* (0.971)*	0.020* (0.683)	0.121 (0.831)*
%ID (Day 8)	0.206 (0.263)	0.124 (0.315)	0.169 (0.255)
Tumor/Liver (Day 8)	0.356 (0.156)	0.380 (0.127)	0.127 (0.125)
Tumor/Tissue (Day 8)	0.159 (0.321)	0.508 (0.093)	0.089 (0.132)

## Discussion

Measuring the uptake of a radiopharmaceutical incorporates several important variables. Accurate measurement of the radiopharmaceutical pre- and post-injection to evaluate injected dosage can be affected by scanner calibrations, decay and dead-time corrections, precise fusion of the anatomical and functional image for attenuation correction, the reconstruction algorithm, and the acquisition time relative to the injection. Imaging calibrated National Institute of Standards and Technology (NIST) phantoms cross-referenced to a dose calibrator using developed and standardized imaging protocol assures accuracy in the PET measurements [22]. The image reconstruction algorithm and the acquisition time interval after injection should be standardized for comparing baseline and post-therapeutic imaging sessions [23].

Distribution of a radiopharmaceutical is also dependent on many biological and functional patient specific parameters: such as the patient’s respiration, drug interactions with the imaging agent, disease state, and the patient’s body height and weight [24]. Tracer uptake calculations have commonly used the activity within a ROI, decay corrected to the time of injection and normalized by the amount injected, which can be corrected for body weight, body surface area, or lean body mass [25] to consider the overall distribution of the radiopharmaceutical within the subject’s body.

Researchers have evaluated many other parameters that affect quantitative analyses, such as differences in the ROI [26], image acquisition, reconstruction, and effect of image noise [14, 24, 27–30] and have implemented quantitative techniques such as SUV peak [6], max, or mean [22] to account for those parameters.

SUVbw(max) is the maximum SUV value of a pixel within the tumor. SUVpeak was implemented in clinical studies to provide a larger standard volume centered on the maximum pixel within the tumor to reduce variations of a limited set of SUV values for comparison in efficacy studies. In clinical studies, SUV(peak) [6] calculates the SUV in a fixed ROI (1.2 cm diameter) centered on the pixel of maximum peak value. The concept of SUV(peak) is to obtain a high Signal-to-Noise Ratio (SNR) within a standard ROI to allow for the comparison of different cohorts, but the standard 1.2 cm ROI is difficult to implement in preclinical PDX studies. Unfortunately, preclinical tumors are enrolled in a PET study at approximately 0.5 cm or less in diameter sothe large SUV(peak) ROI is not suitable for small tumors in mice. To obtain a high SNR for a quantitative method similar to SUVpeak but for preclinical analysis, SUVbw(mean-50%) was employed with the mean of the SUVbw values within a volume of pixels within a 50% threshold of the maximum SUV value, which therefore reduces the statistical variations in a manner similar to SUVpeak.

Adams [24] demonstrated for small tumors that SUV(max) will provide a closer relationship to the physiological tracer uptake concentration than SUV(peak). Another high SNR method pertinent to PET preclinical PDX studies, as demonstrated, is the implementation of SUVbw(mean) with a ROI of a percentage of the maximum value.

In addition to tumor uptake normalized by the injected activity and body weight or surface area or lean body mass, others have evaluated normalizing to the liver [31], other tissue like muscle or non-tumor contralateral side. Park [31] normalized the FDG tumor SUV (max) to the SUV(liver), using a 3 cm diameter circular ROI in the right liver lobe displaying homogeneous FDG uptake. As shown in Fig. 3, both the liver and tissue %ID FLT uptake varied over the course of the drug administration and between vehicle and combination groups, demonstrating that normalization by liver or tissue can be misleading. This technique should be used cautiously in preclinical drug studies. The investigator first needs to establish how consistently drug treatment affects the radiopharmaceutical uptake in the liver over the drug cycles prior to normalization and may need to normalize to a different tissue with less variability in the specific study.

Issues in the present study were two-fold: normalization methods and small sample size in each cohort. A normalization to baseline was applied to each cohort to compare the quantitative methods, which can result in large standard deviations, i.e. %ID, due to the very small FLT tumor uptake which is several orders of magnitude less than the injected activity, while the FLT uptake in the tumor, muscle, and liver was of the same magnitude.

The small sample size (*n* = 5 to 7 per group) is a weakness which limits the study’s statistical power, complicating the detection of differences between the quantitative methods. Comparing the quantitative methods for each cohort and time-point, the power (1-β) (≥0.8 highlighted * and bold), effective size [in brackets], and the total number of samples required to obtain a power of ≥ 0.8 (in parenthesis), based on the standard deviations in the present study, are displayed in Table 6 (G*Power (ver 3.1.9.7) [ref 32]).

**Table 6:** Comparison of the quantitative methods, power (1-β) (values ≥ 0.8 highlighted * and bold), effective size (values in brackets] and the total number of samples required for a power of 0.8 (values in parenthesis) based on the standard deviations in the present study, calculated with G*Power (ver. 3.1.9.7) [ref 32]

Cohort	PDX Model		
	BL0293–F563	BL0479–F1894	765638-272-R
Vehicle: Day 3 or 4	0.370 [0.432] (*n* = 70)	0.793* [0.688] (*n* = 30)	0.399 [0.449] (*n* = 65)
Combination Treated: Day 3 or 4	0.581 [0.553] (*n* = 45)	0.555 [0.538] (*n* = 45)	0.065 [0.108] (*n* = 1000)
Vehicle: Day 8	0.647 [0.591] (*n* = 40)	0.072 [0.13] (*n* = 700)	0.647 [0.591] (*n* = 40)
Combination Treated: Day 8	0.072 [0.13] (*n* = 700)	0.507 [0.511] (*n* = 50)	0.172 [0.283] (*n* = 155)

**Fig. 5:**
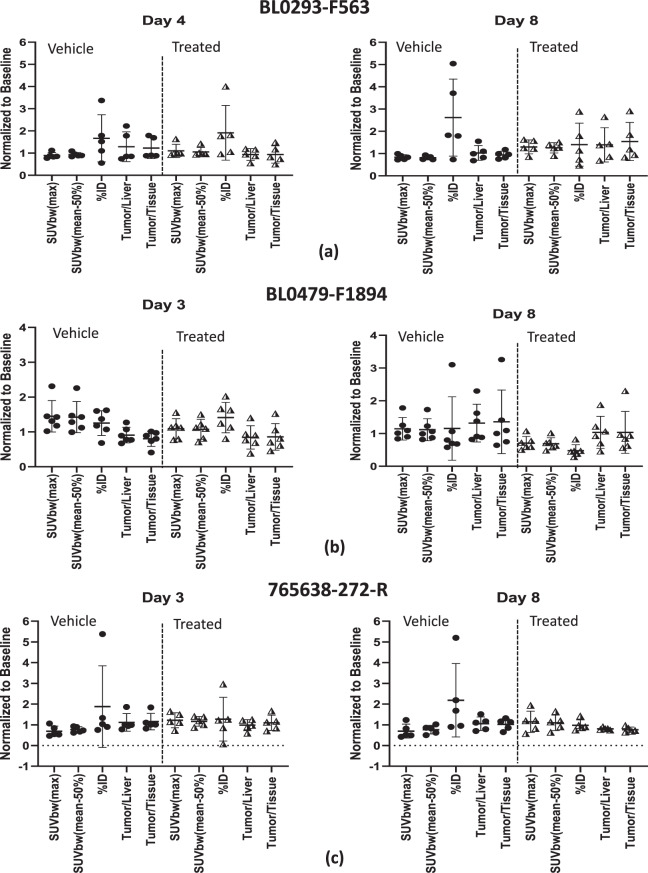
FLT tumor uptake (mean ± sd) normalized to baseline for each analysis method for models (**a**) urothelial/bladder cancer, nos: BL0293–F563, (**b**) transitional cell carcinoma/urothelial cancer: BL0479–F1894, and (**c**) squamous cell lung carcinoma: 765,638-272-R. day 3 or 4 (left panel) and day 8 (right panel): vehicle: circles and treated-combination: triangles

The high power (0.79) for the PDX model BL0479–F1894 for the vehicle cohort at day 3 demonstrated harmonization of the different quantitative methods, while five cohorts demonstrated a power (between 0.5 to 0.7) and six cohorts with a power less than 0.5. The lower power can be attributed to the large standard deviations for the quantitative methods: %ID and tumor to organs (muscle and liver), as shown in Fig. 5. Impractical increases in cohort size would be required to increase statistical power (≥0.8) across the board.

The final limitation was the comparison of the quantitative methods to volumes for evaluation of efficacy rather than the survival end-point due to the IACUC tumor size limitation, and not a biomarker or pathological result.

## Conclusion

In this study, we have demonstrated that among the methods we tested, SUVbw (max) and SUVbw(mean-50%) had greater predictive power than %ID or the ratios tumor-to-liver or tumor-to-tissue (Fig. 5). Additionally, any of the usual semi-quantitative (SUV(max), SUV(peak), SUV(mean), SUV(tumor)/SUV(liver), SUV(tumor)/SUV(tissue), and %ID) techniques can be successfully used as long as the researcher understands the effect of the drug and imaging agent on the normalization parameter (i.e. liver, tissue, body weight, lean body mass). For drug treatment studies, comparison between cohorts such as vehicle and drug or other cohorts (i.e. patients) the SUV calculation should be implemented and tumor activity preferably corrected by body weight or lean body mass or surface area, injected dose, and radioactive decay. Cohort sizes should be as large as practicable to increase the study power.

Variability in imaging protocols and quantitative analysis techniques has hindered the ability to compare preclinical chemotherapy response studies. This study provides the preliminary results to demonstrate that all quantitative methods can be applied to discern comparisons between cohorts and in meta-analysis studies, but the end user should be cognizant that the therapeutic drug(s) can affect the radiopharmaceutical uptake in the organ of interest used for normalization. SUV calculations (peak, mean with a threshold, and max) have a two-fold normalization of the radiopharmaceutical uptake in the tumor, injected dose decayed to time of acquisition and the overall distribution of the radiopharmaceutical within the body, normalized by body weight, or body surface area, or lean body mass. Even though for the quantitative method %ID, the tumor radiopharmaceutical uptake is normalized by the injected dose, it does not account for the overall distribution of the radiopharmaceutical which can lead to large statistical errors.

Even though there are general trends in the quantitative methods with caveats to the type of normalization, any of the quantitative methods can provide a consistent technique for comparison of different studies (meta-analysis). SUV’s (peak, max and mean) should be used for response to a therapeutic treatment due to the two-fold normalization (injected activity and body mass) resulting in lower p-values and higher power [32].

## Electronic supplementary material

Below is the link to the electronic supplementary material.

Supplementary Material 1

## Electronic supplementary material

Below is the link to the electronic supplementary material.

Supplementary Material 2

## Electronic supplementary material

Below is the link to the electronic supplementary material.

Supplementary Material 3

## Data Availability

The datasets used and/or analyzed during the current study are available in the supplementary materials (S3) section.

## Abbreviations

PET: Positron Emission Tomography
FLT: 3’-deoxy-3’-[F-18] fluorothymidine
SUV: Standard Uptake Value
%ID: Percent Injected Dose
PDX: Patient Derived Xenograft
ROI: Region of Interest
CT: x-ray computed tomography
IACUC: Institutional Animal Care and Use Committee
SNR: Signal-to-Noise Ratio
