# Supplementary material for: Comparison of quantitative imaging analysis methods to evaluate murine [18F]FLT PET therapy response studies
Source: J Transl Med. 2025 Nov 26;24:8. doi: 10.1186/s12967-025-07475-2 (PMC12763824; doi:10.1186/s12967-025-07475-2)
Supplement: Supplementary file 1 — Supplementary Material 1 [file 12967_2025_7475_MOESM1_ESM.pdf]

SOP50102: PDX Implantation, Expansion and Cryopreservation (Subcutaneous)

Effective Date: 6/25/2020

Please check for revision status of the SOP at

https://pdmr.cancer.gov/sops/

PDMR NCI Patient-Derived Models Repository
An NCI Precision Oncology Initiative℠ Resource

TABLE OF CONTENTS

1.0 PURPOSE/SCOPE 3
2.0 SAFETY 3
3.0 CLEAN-UP 3
4.0 EQUIPMENT 4
5.0 GENERAL WORKFLOW FOR TISSUE RECEIPT AND BANKING 6
6.0 TISSUE PREPARATION 7
7.0 IMPLANTATION 8
8.0 GROWTH ASSESMENT 8
9.0 COLLECTION OF QC MATERIAL 10
10.0 CRYOPRESERVATION OF PDX MATERIAL 10
11.0 PASSAGING INTO ALTERNATE HOST STRAINS 12
APPENDIX 1: TUMOR GROWTH SUPPLEMENTS AND ALTERNATE IMPLANT METHODOLOGIES 13
REFERENCES 15

VERSION INFORMATION

1. Change History

Revision	Description
4/17/2017	New Document for PDMR Public Website
6/28/2018	Separated Patient tissue tumor implantation and PDX fragment passaging and cryopreservation into two separate SOPs (SOP50101 and SOP50102, respectively). Updated recommendations on implantation from cryopreserved material, updated company/catalog numbers
6/25/2020	Add additional basement membrane extract options for implantation. Updated syringe-type details.

2. Related SOPs

SOP20101: Fresh Tumor Collection and Handling for Generation of Patient-Derived Models
SOP50101: Patient Tumor Implantation (Subcutaneous) and Monitoring
SOP50103: Histopathological Assessment of Patient-Derived Xenografts

1.0 PURPOSE/SCOPE

This Standing Operating Procedure (SOP) describes the procedures for tissue preparation and procedures for subcutaneous implantation to generate patient-derived xenografts (PDXs) and cryopreservation of material under BSL-2 safety criteria.

This SOP is used/performed by the Biological Testing Branch (BTB) at NCI-Frederick, Frederick National Laboratory for Cancer Research.

2.0 SAFETY

BTB treats all patient tissue and mice carrying patient tumors as a possible health threat as the human tissue could still retain human pathogenic agents. Mice are housed in barrier facilities using full micro-isolator technique with all work, including husbandry, provided by experienced technical staff. The primary mouse strain used is the NOD.*Cg-Prkdc*^{scid}*Il2rg*^{tm1Wjl}/SzJ (NSG) which are highly susceptible to infection due to their profound immunodeficiency. All materials coming into the barrier facilities are decontaminated by autoclaving, or chemical means including the use of chlorine dioxide gas, Clorox Healthcare Hydrogen Peroxide spray/wipes, and Virkon® dip tanks for non-autoclavable items.

3.0 CLEAN-UP

3.1 All materials in contact with patient tissue, as well as the mice carrying patient tumor samples and cultures derived from patient tumor samples, are treated as a potential health threat (BSL-2 precautions) since the human tissues could retain human pathogenic agents even if they do not replicate in mouse cells (e.g., EBV, HPV, etc).

3.2 Flush/soak any items (e.g., tubes, syringes, petri dishes, lab mats, etc) that were in contact with human tissue with disinfectant (e.g., 10% bleach, commercial hydrogen peroxide disinfectant, 2% Virkon®) for a minimum of 10 minutes before disposal in biohazard waste or sharps containers (follow institutional guidelines and manufacturer's recommendations).

3.3 For items that can't be rinsed (e.g., micropipettors), wipe down thoroughly with bleach-soaked gauze or other appropriate disinfectants.

4.0 EQUIPMENT

Review all equipment based on method of interest

4.1 Implant Materials & Equipment

4.1.1 200 µL pipettor and sterile tips

4.1.2 Sterile scalpel and forceps

4.1.3 Sterile petri dishes

4.1.4 Matrigel® (BD BioSciences, Bedford, MA.) or Cultrex™ Basement Membrane Extract Type 3 (BME3), Pathclear, (R&D Systems, Cat#: 3632-001-02)

- IMPORTANT: All basement membrane extract purchases should be submitted specifying mouse rodent pathogen screened LDEV-Negative material. If not, there is a possibility of LDEV contamination which can result in LDEV+ tumors.

4.1.5 11-gauge Trocar (tissue implant needle), sterile (pre-chilled)

4.1.6 1-cc low dead space syringe with luer-lock hub, sterile (pre-chilled at -20°C) (Exel Cat#: 26048-1)

4.1.7 RPMI-1640 containing primocin (working concentration: 100 µg/mL)

4.1.8 NOD.*Cg-Prkdc*^{scid}*Il2rg*^{tm1Wjl}/SzJ (NSG) mice, sex-matched to human patient

4.1.9 Isoflurane and anesthesia machine for delivery

4.1.10 Marcaine solution, 0.25% (Bupivacaine HCl)

4.1.11 Nolvasan® (disinfectant for animal surgery)

4.1.12 Clorox Healthcare® Hydrogen Peroxide Spray, Bleach (5.25% Hypochlorite) diluted 1:10, 2% Virkon®, or similar disinfectant

4.2 Alternate Implant Methodology: Additional Materials

4.2.1 Estradiol pellets (Innovative Research; 3.0-mm, 90-day release, 0.18 mg)

4.2.2 Testosterone pellets (Innovative Research; 3.0-mm, 90-day release, 12.5 mg)

4.2.3 10X PBS, sterile

4.2.4 Sterile water

4.2.5 10 mm cloning ring, sterile (Sigma Catalog# C2059-1EA)

4.2.6 6-well plate, sterile

4.2.7 Advanced DMEM/F12 containing 5% fetal bovine serum (FBS) and primocin (working concentration: 100 µg/mL)

4.2.8 Wound closure: sterile staples or sutures

4.3 Cryopreservation Materials & Equipment

4.3.1 Pipetman, 1000 µL and sterile tips

4.3.2 Sterile scalpel, forceps and scissors

4.3.3 Sterile petri dish

4.3.4 RPMI-1640 media containing 20% fetal bovine serum (FBS) and 10% DMSO (Burdick&Jackson Brand); made same day as use

4.3.5 2-mL Cryovials and tube holder

4.3.6 Tumor material freshly harvested from PDX-bearing mice RPMI-1640 with primocin, maintained at 4ºC by placing in sterile petri dish on wet ice

4.3.7 Bucket of ice (wet not dry)

4.3.8 Stepped Rate Cryopreservation Freezer or an alcohol-free cell freeze container (e.g., CoolCell) or isopropanol-based (e.g., Mr. Frosty) freeze container can also be used for slow-rate freezing if a stepped rate freezer is not available.

5.0 GENERAL WORKFLOW FOR TISSUE RECEIPT AND BANKING

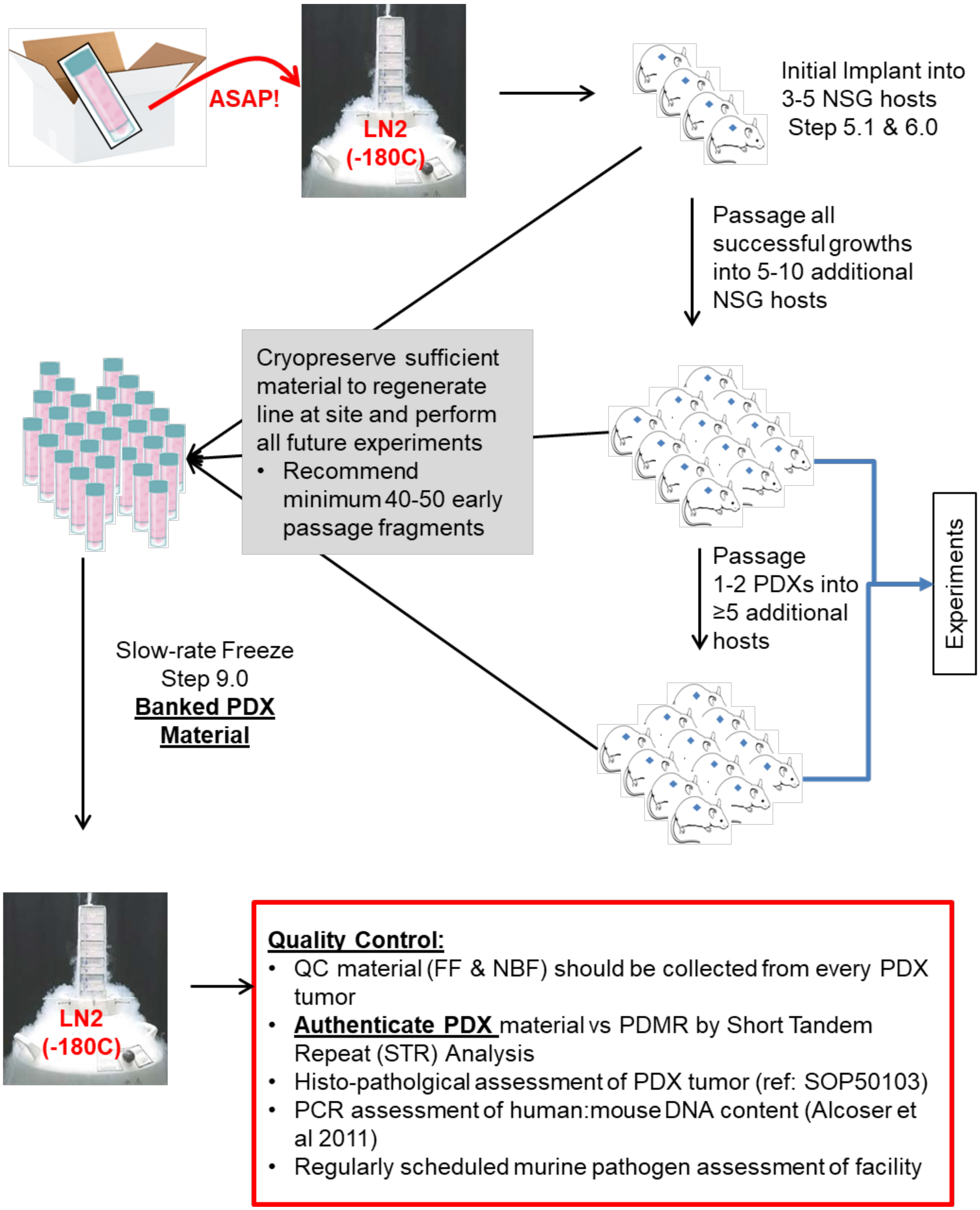

6.0 TISSUE PREPARATION

- All tissue preparation should be performed in a Biological Safety Cabinet (BSC) using sterile instruments and technique. Maintain sterility at all times.
- ***Important:*** All set-up for implantation should be in place prior to beginning to thaw the cryopreserved tissue fragments. Once the cryopreserved tissue fragments are removed from the freezer, the following steps should proceed quickly.

6.1 Cryopreserved PDX Fragment

6.1.1 Remove cryovial from liquid nitrogen, transfer to lab on dry ice, and hold until ready to implant.

6.1.2 When ready to thaw, swirl the tube in a 37°C water bath until the contents are almost completely melted.

6.1.3 Spray/wipe outside of cryovial with disinfectant, then move the vial into a BSC.

6.1.4 Transfer PDX fragments into a petri dish containing fresh RPMI-1640 medium to rinse/dilute the DMSO-containing freeze media away.

6.1.5 Using a sterile scalpel cut the tissue into 1-2 mm^3 fragments (~30 mg).

6.1.6 Place each tumor fragment into the end of an 11-gauge Trocar needle and add a drop of Matrigel®/BME3 (~50-100 µL).

6.1.7 Keep Trocar on ice to prevent Matrigel®/BME3 from polymerizing until implantation.

6.2 Freshly harvested PDX fragments

6.2.1 Transfer the PDX material into a sterile petri dish containing fresh DMSO-free RPMI-1640 medium to keep the tissue wet.

6.2.2 Using a sterile scalpel cut the tissue into 1-2 mm^3 fragments (~30 mg).

6.2.3 Evaluate the material prior to cutting to ensure tumor tissue is being implanted as non-tumor tissue (e.g., cartilage, muscle) can provide supporting cells but should not be implanted separately. Grossly necrotic areas are avoided.

6.2.4 Place each tumor fragment into the end of a pre-chilled 11-gauge Trocar needle and add a drop of Matrigel®/BME3 (~50-100 µL).

6.2.5 Keep the Trocar on ice to prevent the Matrigel®/BME3 from polymerizing until implantation.

7.0 IMPLANTATION

NCI PDMR nomenclature for counting of passaged material: Patient material is referred to as Originator and the PDX grown in the first implanted host NSG mouse is called passage 0 (P0). Subsequent passages are iterative. Each PDX/host mouse identifier is a unique identifier which includes the parental identifiers so that the full passage lineage can be traced back to the Originator material.

Protocol for use of tumor growth supplements and alternate implant methodologies (mammary fat pad [breast cancer], thigh muscle [sarcomas]) can be found in Appendix 1.

7.1 Subcutaneous Implantation Procedure

7.1.1 Shave the implantation site prior to anesthesia.

7.1.2 Anesthetize a sex-matched NSG mouse by isoflurane inhalation until non-responsive to toe-pinch. Keep mouse warm and monitor breathing during procedure.

7.1.3 Disinfect implantation site with Nolvasan®. We recommend to NOT use alcohol, as this can chill the animal during the procedure.

7.1.4 Using scissors, make a small nick incision in the inguinal area (for standard SC implants placed in the posterior axillary area) and insert the Trocar through the skin opening.

7.1.5 Slide the Trocar craniodorsally to about 0.5 cm caudal to the axillary area; the Trocar should slide smoothly under the skin. Expel the tumor fragment into the axillary area. This may require twisting the Trocar slightly to ensure the tumor fragment does not stick to the Trocar and then gently pull out the Trocar. Forceps can be used over the skin to hold the fragment in place during trocar withdrawal.

7.1.6 The entry site will heal well and does not require a wound clip, although one may be applied if preferred.

7.1.7 Apply 1-2 drops 0.25% Marcaine to the nick incision to relieve pain/discomfort.

7.1.8 Keep mice warm and monitor breathing until awakened from anesthesia.

8.0 GROWTH ASSESMENT

8.1 Observation

8.1.1 Mice are weighed 1-3 times per week as a gauge of animal health and more frequently if clinical signs are noted.

8.1.2 Mice are observed at least once daily and tumor growth is monitored with caliper measurements at least once weekly. The tumor weight (mg) is calculated using the following formula [3]:

$$Tumor\ weight\ (mg) = \frac{(tumor\ length)x\ (tumor\ width)^2}{2}$$

8.1.3 P0 (first passage in mouse) PDX host mice are monitored for up to 300 days after implantation at the NCI PDMR. Mice not showing progressive tumor growth by 300 days post-implant are euthanized.

8.1.4 Antemortem determination for harvesting tumor tissues is based upon tumor size as well as clinical signs. Animals without palpable tumors are evaluated clinically and by monitoring body weights.

8.2 Tumor Assessment and Humane Euthanasia

8.2.1 Tumors are harvested immediately following euthanasia or under terminal isoflurane anesthesia.

8.2.2 Palpable tumors are evaluated 2-3 times per week and efforts are made to identify animals that may experience adverse health effects before the next observation (and euthanize accordingly).

8.2.2.1 It is recommended to harvest PDX tumors in the 1000-2000 mg size range as there is typically less necrotic tissue present. (Individual institutional guidelines for maximum allowable tumor burden should be followed)

8.2.2.2 An animal with a tumor that grows in a manner that limits their mobility, impedes access to food and water, or has a tumor that is showing the onset of skin necrosis is euthanized.

8.2.2.3 Tumors may be red to purple in color when highly vascularized and this appearance does not warrant euthanasia. When the skin overlying the tumor shows evidence of necrosis or other compromise, euthanasia is warranted.

8.2.2.4 Animals with tumors arising at sites other than the implantation site will be euthanized based mainly upon gross observations such as weight loss or gain, lethargy, respiratory distress, inactivity, ruffled hair coat, decreased body temperature, inappetance, etc.

8.2.2.5 Some internal tumors (prostate, spleen, renal) may be palpable and will be checked regularly so that the tumor does not become large and debilitating.

8.3 Static and No-Growth Monitoring

8.3.1 Animals with no signs of tumor growth are euthanized at 300 days post-implant.

8.3.2 NCI has observed numerous tumors that we have categorized as "Static" tumors.

8.3.2.1 These tumors reach a steady-state tumor burden and appear to stop growing in size; possibly due to walling off of the tumor in the animal.

8.3.2.2 These mice are euthanized and fragments of the tumor are passaged to determine if tumor growth will re-initiate.

9.0 COLLECTION OF QC MATERIAL

9.1 The NCI PDMR recommends collecting a minimum of 1 NBF fragment and 3-5 flash frozen fragments from every PDX for quality control and validation material.

9.1.1 Flash-frozen PDX fragments

9.1.1.1 Snap freeze PDX fragment in a cryovial on in LN2 immediately upon collection to preserve analytes. Store at -80C.

9.1.1.2 End uses: (1) DNA for short tandem repeat (STR) assessment, (2) DNA for human:mouse DNA ratio assessment, (3) DNA and RNA for NextGen sequencing, (4) protein extraction for RPPA analysis, (5) human and murine pathogen assessment.

9.1.2 NBF fragment

9.1.2.1 (1) Formalin-fixed, paraffin-embedded fragment sent for histo-pathological assessment and immunohistochemistry assessment.

9.2 Note that since these are single fragments from a human tumor growing in a mouse host and different PDX tumors can have different stromal content, it is possible to collect fragments that are 100% mouse stroma. QC fragments should be collected from areas that are by gross examination not necrotic and appear to be tumor.

10.0 CRYOPRESERVATION OF PDX MATERIAL

10.1 Tumor preparation for cryopreservation.

10.1.1 In a BSC, harvest the tumor into a sterile petri dish containing a small volume of RPMI-1640 with primocin.

10.1.2 Using a sterile scalpel, cut the tissue into ~2-3 mm^3 fragments (~30mg).

10.1.3 With the cryovials in a tube rack held on ice, add 3-5 fragments to each tube then add 1-mL of freeze medium (RPMI 1640/5% FBS/10% DMSO) to each tube without exceeding the tube fill volume.

10.1.4 Apply the cap to the cryovials, seal well. Wipe the exterior with disinfectant then place into wet ice until ready to begin stepped--rate freezing.

10.2 Stepped -Rate Cryopreservation Procedure

10.2.1 Mechanically controlled stepped-rate freeze cryopreservation is recommended, when available, as it is believed to result in a lower loss of cell viability due to the decreased formation of ice crystals.

10.2.2 Always follow the manufacture’s guidelines for operation. General stepped-rate cryopreservation parameters used at by the PDMR are:

- Decrease 1°C/minute down to -4°C
- Decrease 25°C/minute down to -40°C
- Increase 15°C/minute up to -12°C
- Decrease 1°C/minute down to -40°C
- Decrease 10°C/minute down to -90°C

10.2.3 Transfer the cryopreserved vials to liquid nitrogen storage.

10.3 Slow-rate freezing (isopropanol-based using a cryo 1°C cell-freezing container such as Mr. Frosty)

10.3.1 Follow the manufacturer’s instructions as provided for the specific cryopreservation device.

10.3.1.1 PDX material should be held on wet ice in cryovial tubes until ready for placement into the slow-rate freeze container.

10.3.1.2 The base of the cryo-container is filled with isopropanol per the manufacturer’s recommendation and the tube holder is placed on top.

10.3.1.3 Transfer the cryovials filled with fragments/freeze media from the ice-bucket into the tube holder of the cryo-container, screw the lid securely onto the cryo-container, and place at -80°C for a minimum of 4 hrs, but most commonly for overnight.

- If the tubes are held on ice, the cryo-container can be at refrigerator temperatures. If the cryovials are processed at room temperature rather than 4°C, then the cryo-container should be at room temperature.

10.3.1.4 Vials should be transferred to the vapor phase of a liquid nitrogen tank as soon as practical after the 4-hr freeze step, typically the following morning. In no case, should the vials be held longer than 3 days at -80°C before transfer into the vapor phase of a liquid nitrogen storage tank.

10.4 Slow-rate freezing (non-isopropanol based such as CoolCell)

10.4.1 Follow the manufacturer’s instructions as provided for the specific cryopreservation device.

11.0 PASSAGING INTO ALTERNATE HOST STRAINS

11.1 Once the initial vial received from the PDMR has been successfully grown, transplanted, and sufficient viably cryopreserved vials have been banked, growth in other host strains can be attempted.

11.2 We recommend at passaging, instead of passaging into 5 NSG mice, passage into 3 NSG and 2 alternate host strains such as athymic nude. For models with lower take-rates you may want to plan for 10 implants with a similar distribution.

11.2.1 Note you will have an increased chance of success passaging to a new strain if this is done while the tumor is in active passage. Growth characteristics and ability to regrow from a cryopreserved fragment for PDXs grown in the alternate strain will need to be determined independently of the expected growth characteristics determined by the PDMR for the same model grown in NSG mice.

11.3 Banking of PDX material should track the host strain used for purposes of future molecular analysis.

APPENDIX 1: TUMOR GROWTH SUPPLEMENTS AND ALTERNATE IMPLANT METHODOLOGIES

1. Tumor Growth Supplements

A. Tumor types that may be dependent upon exogenously administered hormones (e.g., breast, prostate, ovarian, testicular) should receive subcutaneously implanted hormone pellets to aid in tumor survival and growth.

B. Estradiol pellets (Innovative Research; 3.0-mm, 90-day release, 0.18 mg) should be implanted subcutaneous into mice receiving tissue implants originating from female patients with cancer of the Breast, Ovary, Uterus, Fallopian tube, or Cervix unless contra-indicated in patient diagnoses record.

   a. A single pellet (or 2 if desired) used in an NSG mouse should be adequate to provide the needed support for tumor growth and survival while avoiding estrogen toxicosis. Only one pellet should be used in athymic nude mice due to their sensitivity to estrogen toxicosis.

   b. Injectable estradiol cypionate is avoided if possible as it is more likely to result in estrogen toxicosis before the tumor is able to grow.

   c. Implant pellet at a SC site distant to the PDX implant. The pellet should be replaced every 90 days.

C. Testosterone pellets (Innovative Research; 3.0-mm, 90-day release, 12.5 mg) should be implanted subcutaneously into mice receiving tissue implants originating from male patients with cancer of the testicles, prostate, vas deferens, and zeminal vesicles unless contra-indicated in patient diagnoses record.

   a. A single pellet in an NSG mouse should be adequate to provide the needed support for tumor growth and survival.

   b. Implant pellet at a site distant to the PDX implant. The pellet should be replaced every 90 days.

2. Common PDMR Alternate Implant Methodologies

PDMR implants essentially all tumor types subcutaneously (SC) per the method above except breast tumors which are implanted in the mammary fat pad (MFP).

Adult Soft Tissue Sarcomas are implanted using the "Sarcoma Plug" method below as well as SC (if sufficient material) at P0. P1 tumors are then implanted at the alternate site as well as SC to determine if SC growth is supported. For adult soft tissue sarcomas, the PDMR noted a low take-rate with direct SC implantation of material. The "Sarcoma Plug" method has increased take-rate from <5% to 35%-40% across all sarcoma histologies.

Other alternate implant methodologies are used less frequently and will be posted as a separate SOP to the PDMR website. After successful growth in the alternate implant site, PDMR may try growth SC and if successful do subsequent passages there.

A. "Sarcoma Plug" Implant [1, 2]

a. The Day Before Implantation:

i) Using a sterile forceps, press a sterile 10-mm cloning ring, greased side down, in the center of a well in a sterile 6-well plate to create a seal between the plate and the ring.

ii) Quickly layer ~100 µL of Matrigel®/BME3 into the cloning ring followed by addition of a small tumor fragment. Cover the fragment with an additional~ 100 µL of Matrigel®/BME3.

iii) Add adequate media (Advanced DMEM/F12 with 5% FBS and primocin) to fill the cloning ring and incubate overnight in a 37°C CO_2 incubator. If the ring must be incubated longer than 24-h, then the entire well should be filled with media such that the cloning ring is covered to prevent dessication.

b. Day of Implantation

i) Using a sterile forceps, remove the cloning ring from the 6-well plate and sterilely remove the "Sarcoma Plug" from the cloning ring. We have found using the end of a sterile 1-cc plunger works well to expel the plug from the cloning ring.

ii) Follow the same mouse preparation procedures as that used for SC implantation with the following changes:

- Make a blunt dissection just cranial to the rear leg for access to the #4 (#9) MFP.
- Use a small spatula to "scoop" up the "Sarcoma Plug" and place it into the subcutis of a surgically anesthetized mouse, near but not within the MFP.

iii) Close the skin and staple (or suture if preferred) the overlying skin

iv) Apply 1-2 drops 0.25% Marcaine to the nick incision to relieve pain/discomfort.

v) Keep mice warm and monitor breathing until awakened from anesthesia.

B. Mammary Fat Pad (MFP) Implant

a. Follow the same mouse preparation procedures as that used for SC implantation with the following changes:

i) Make a small incision just cranial to the rear leg for visualization of the #4 (#9) MFP.

ii) Gently lift the MFP with forceps, incise the gland creating a small pocket. Slide the Trocar into this space and expel the prepared tumor fragment into the pocket.

iii) Return the gland to its proper position, close and staple (or suture if preferred) the overlying skin.

iv) Apply 1-2 drops 0.25% Marcaine to the nick incision to relieve pain/discomfort.

v) Keep mice warm and monitor breathing until awakened from anesthesia.

REFERENCES

1. Kenney, S., et al., *ASPS-1, a novel cell line manifesting key features of alveolar soft part sarcoma.* J Pediatr Hematol Oncol, 2011. **33**(5): p. 360-8.
2. Vistica, D.T., et al., *Therapeutic vulnerability of an in vivo model of alveolar soft part sarcoma (ASPS) to antiangiogenic therapy.* J Pediatr Hematol Oncol, 2009. **31**(8): p. 561-70.
3. Plowman J, D.D., Hollingshead M, et al., *Human tumor models in NCI drug development*. Anticancer Drug Development Guide: Preclinical Screening, Clinical Trials, and Approval. 1997.
