# Supplementary material for: Comparison of quantitative imaging analysis methods to evaluate murine [18F]FLT PET therapy response studies
Source: J Transl Med. 2025 Nov 26;24:8. doi: 10.1186/s12967-025-07475-2 (PMC12763824; doi:10.1186/s12967-025-07475-2)
Supplement: Supplementary file 2 — Supplementary Material 2 [file 12967_2025_7475_MOESM2_ESM.pdf]

Table of Contents

CHANGE HISTORY 1
RELATED SOPS 1
1.0 PURPOSE/SCOPE 2
2.0 SAFETY 2
3.0 CLEAN-UP 2
4.0 EQUIPMENT 2
5.0 ANIMAL PREPARATION 3
6.0 ANIMAL MONITORING DURING IMAGING PROCEDURE 4
7.0 CT IMAGING PROCEDURE 4
8.0 PET IMAGING PROCEDURE 4
9.0 POST-IMAGING PROCEDURE 5
10.0 PET/CT IMAGE FUSION AND DATA ANALYSIS PROCEDURE 5
APPENDIX 1: EXTENDED METHODOLOGY 5

CHANGE HISTORY

Revision	Description
	Internal SOP used by SAIP Laboratory
12/01/2019	Standardize SOP for posting to PDMR-TCIA Public website

RELATED SOPS

1.0 PURPOSE/SCOPE

The Standard Operating Procedure (SOP) describes the procedures for animal handling and monitoring, anesthesia, daily PET/CT QA, PET/CT acquisition and anlaysis for detection and monitoring of tumors. This SOP is used/performed by the Small Animal Imaging Program (SAIP) at NCI-Frederick, Frederick National Laboratory for Cancer Research.

2.0 SAFETY

SAIP treats all patient tissue and mice carrying patient tumors as a possible health threat as the human tissue could still retain human pathogenic agents. Mice are housed in disposable cages located barrier facilities, handled according to ABSL1 procedures, provided by experienced technical staff. The primary mouse strain used is the NOD.*Cg-PrkdcscidIl2rgtm1Wjl*/SzJ (NSG) which are highly susceptible to infection due to their profound immunodeficiency. All materials coming into the barrier facilities are decontaminated by autoclaving, or chemical means for non-autoclavable items. Mice are transported in their cages to the imaging scanners which are located within the barrier facility. Anesthesia is exhausted from nose cones and induction chamber according to local regulations (carbon filter or active system).

All personnel working with radioactive material received specialized training as per the local and federal guidelines.

3.0 CLEAN-UP

3.1 All materials coming into contact with patient tissue as well as the mice carrying patient tumor samples are treated as a potential health threat (BSL-2 precautions) since the human tissues could retain human pathogenic agents even if they do not replicate in mouse cells (e.g., EBV, HPV, etc).

3.2 Flush/soak any items (e.g., tubes, syringes, petri dishes, lab mats, etc) that were in contact with human tissue with disinfectant (e.g., 10% bleach, Cavicide®, commercial hydrogen peroxide disinfectant, 2% Virkon®) before disposal in biohazard waste or sharps containers (follow institutional guidelines and manufacturer's recommendations).

3.3 For items that can't be rinsed, wipe down thoroughly with bleach-soaked gauze or other appropriate disinfectants.

4.0 EQUIPMENT

4.1 Dose, Image Acquisition Equipment and Analysis software

4.1.1 CRC-15R Dose Calibrator (Capintec Inc., Florham Park, NJ)

4.1.2 Inveon micro-PET/CT scanner (Siemens Medical Solutions USA, Inc., Knoxville, TN)

4.1.3 MIM workstation software version 6.6.5 (MIM Software Inc., Cleveland, OH)

4.2 Animal Model, Handling and Monitoring Materials & Equipment

4.2.1 Isoflurane, Anesthesia-vaporizer, Anesthesia Induction chamber

4.2.2 Vented hood

4.2.3 Temperature regulated imaging bed and monitoring system to maintain animal body temperature (37 ± 0.5 °C).

4.2.4 Pulmonary pad and respiratory monitoring system

4.2.5 Tumor bearing NOD.*Cg-Prkdc*$_{scid}$*Il2rg*$_{tm1Wjl}$/SzJ (NSG) mice, sex-matched to human patient (PDM SOP50101).

4.3 Personal Protective Equipment

4.3.1 Personal Protective Equipment (PPE) at a minimum laboratory scrubs, latex or nitrile glove (2 pair), hair bonnet, facility shoes, and safety glasses.

4.3.2 Anesthesia is vented according to regulations.

5.0 ANIMAL PREPARATION

5.1 Mice are to be fasted for 10-12 hours prior to injection, with free access to water, for an [^{18}F]-FDG imaging study. Fasting is not required for an [^{18}F]-FLT imaging study.

5.2 Transport cages from vivarium to imaging room and place the cage of mice on a heated blanket (36 - 37 °C) covered with bench coat.

5.3 Warm the mice under the heat lamp for 3-4 minutes prior to i.v. tail vein injection of the radiotracer.

5.4 Injection (IV tail vein) of the mouse is performed in a vented hood.

5.4.1 Measure (dose calibrator) the syringe and residual doses with corresponding times and time of injection.

5.4.2 Immediately after IV injection, anesthetize the mouse via a nose cone administration of 3% isoflurane with oxygen as the carrier gas at 2 liter/minute (lpm) flow rate to reduce [^{18}F]-FDG muscle uptake. The oxygen flow rate is set to 2 lpm since the vaporizer is connected to the nose cone and to pre-load the induction chamber. The induction chamber is located within a vented hood and placed on a heating pad to maintain animal body temperature between 36°C - 37°C while the mouse is anesthetized (uptake phase). After the mouse is anesthetized via the nose cone administration, place the mouse in the induction chamber and lower the isoflurane vaporizer to 1.5 %, shut off valve to nose cone, and lower the oxygen flow rate to 1 lpm.

5.4.3 Mice injected with [^{18}F]-FLT are returned to their cage and are allowed free movement and access to food and water. The cage is placed behind a lead wall to reduce radiation dose to laboratory personnel. Place mice in anesthesia induction chamber 5 minutes prior to scan (2% isoflurane; oxygen flow (1 lpm).

5.5 Prior to transport of mice to the PET/CT scanner, set the scanner isoflurane vaporizer to 2% with oxygen as the carrier gas with a flow rate of 1 lpm.

5.6 Perform the toe pinch test to assure mice are anesthetized, then transport a mouse to the imaging bed (lined with the bench coat). Repeat for second mouse if performing multi-mouse imaging.

5.7 Mice are placed on the temperature regulated heated imaging table in the prone position. Use a respiratory pad to monitor a mouse pulmonary function.

5.8 Follow the manufacturers method to align the mice in the field of view and perform the PET/CT acquisitions (parameters provided below: sections 7 and 8.

5.8.1 PET DICOM Header injection parameters

5.8.1.1 When two mice are imaged simultaneously, the radioactivity entered into the DICOM header is the activity of each mouse decayed to the injection time of the second mouse and summed for both mice. This activity is entered in the syringe dose field and residual dose is zero. All times entered should be the injection time of the second mouse.

5.9 Repeat steps 5.1-5.9 for the remaining mice.

6.0 ANIMAL MONITORING DURING IMAGING PROCEDURE

6.1 Animal monitoring

6.1.1 Animal body temperature is maintained at 37°C ± 1°C using the heated animal imaging bed.

6.1.2 The animals' respiration is monitored by a pulmonary pad / physiology monitoring system and the animal's percent isoflurane is modified to maintain a respiration rate in the range: 50-90 bpm.

7.0 CT IMAGING PROCEDURE

7.1 QA: per manufacturer's protocol.

7.2 CT acquisition parameters:

7.2.1 Voltage: 80 kVp

7.2.2 Current: 500 μA

7.2.3 Projections: 120 steps covering 220-degrees

7.2.4 Exposure time: 200 millisecond per step

7.3 CT image reconstruction parameters:

7.3.1 Reconstruction algorithm: FeldKamp

7.3.2 Image matrix: 192 x 192

7.4 X-ray CT image converted to Hounsfield units (HU) per manufacturers protocol for attenuation correction.

8.0 PET IMAGING PROCEDURE

8.1 QA: per manufacturer's protocol.

8.2 PET acquisition parameters:

8.2.1 Data acquisition mode: list-mode

8.2.2 Energy window: 350–650 keV

8.2.3 Coincidence timing window: 3.432 ns

8.2.4 Acquisition time (min): 20

8.3 PET image reconstruction parameters (scatter and attenuation corrected)

8.3.1 Histogram:

8.3.1.1 # of projections (128); # of angles (160); Span (3); Maximum Ring Difference (79)

8.3.2 Reconstruction algorithm: Ordered Subset Expectation Maximization (OSEM-3D)

8.3.2.1 Subsets: 12

8.3.2.2 Number of iterations: 4

8.3.2.3 Image matrix: 256 x 256

9.0 POST-IMAGING PROCEDURE

9.1 At the conclusion of an imaging session

9.1.1 Move the PET/CT image bed out of the scanner bore to access the mice.

9.1.1.1 Remove mouse from imaging bed and remove pulmonary pad.

9.1.2 Transport the mice to their respective cage with feed and water located on warming pad (maintained at 36°C and 37°C) behind lead shielding. Monitor their breathing until they completely recovered from anesthesia.

9.1.3 Transport cages to their rack located in the animal holding room.

10.0 PET/CT IMAGE FUSION AND DATA ANALYSIS PROCEDURE

10.1 Use MIM workstation software to visualize, co-register PET and CT images, and analyze PET radiopharmaceutical uptake.

10.2 Maximum Standard Uptake Value (normalized by body weight and dosage percent injected): SUVbw max

10.2.1 Utilizing a 3D spherical region-of-interest (ROI), locate the maximum activity (Bq) within the tumor.

10.2.2 Calculate the injected activity [Bq] for the specified mouse.

10.2.2.1 syringe minus residual decay corrected to time of injection.

10.2.3 Calculate SUVbw max: xenograft radiotracer maximum uptake [Bq] normalized by animal body weight (gm) and injected activity [Bq] for the specified mouse of interest.

APPENDIX 1: EXTENDED METHODOLOGY

Additional methodology, reference images, or bibliographic references outside of the details in the SOP can be inserted here
