# Supplementary material for: Comparison of quantitative imaging analysis methods to evaluate murine [18F]FLT PET therapy response studies
Source: J Transl Med. 2025 Nov 26;24:8. doi: 10.1186/s12967-025-07475-2 (PMC12763824; doi:10.1186/s12967-025-07475-2)
Supplement: Supplementary file 3 — Supplementary Material 3 [file 12967_2025_7475_MOESM3_ESM.docx]

| **BL0293-F563** | |  |  |  |  |  |  |  |  |  |  |  |  |  |  |  |  |
| --- | --- | --- | --- | --- | --- | --- | --- | --- | --- | --- | --- | --- | --- | --- | --- | --- | --- |
|  | |  | **SUVbw(max)** | | | **SUVbw(mean-50%)** | | | **%ID** | | | **Tumor/Liver** | | | **Tumor/Tissue** | | |
| **M#** | **Group** | | **Day 0** | **Day 4** | **Day 8** | **Day 0** | **Day 4** | **Day 8** | **Day 0** | **Day 4** | **Day 8** | **Day 0** | **Day 4** | **Day 8** | **Day 0** | **Day 4** | **Day 8** |
| 1274 | Vehicle | | 1.67 | 1.28 | 1.20 | 1.02 | 0.86 | 0.84 | 0.150 | 0.506 | 0.756 | 0.63 | 0.53 | 0.42 | 0.94 | 0.85 | 0.70 |
| 1279 | Vehicle | | 3.25 |  |  | 2.15 |  |  | 0.649 |  |  | 1.54 |  |  | 1.49 |  |  |
| 1280 | Vehicle | | 3.47 | 2.96 | 3.12 | 2.33 | 2.04 | 1.99 | 0.347 | 0.381 | 0.631 | 0.77 | 1.38 | 0.77 | 1.19 | 2.01 | 0.97 |
| 1281 | Vehicle | | 3.50 | 3.91 | 3.47 | 2.10 | 2.29 | 1.53 | 0.501 | 0.759 | 1.859 | 0.97 | 0.83 | 0.80 | 1.38 | 1.22 | 1.33 |
| 1291 | Vehicle | | 3.73 | 3.30 | 2.78 | 2.47 | 2.20 | 1.83 | 0.905 | 0.494 | 0.665 | 0.83 | 0.61 | 0.92 | 1.22 | 1.08 | 1.21 |
| 1296 | Vehicle | | 2.86 | 2.37 | 2.37 | 1.65 | 1.57 | 1.53 | 0.488 | 0.875 | 0.874 | 0.64 | 1.43 | 1.00 | 1.02 | 1.83 | 1.21 |
| 1267 | TMZ/VX-970 | | 1.23 |  |  | 0.83 |  |  | 0.238 |  |  | 0.31 |  |  | 0.41 |  |  |
| 1273 | TMZ/VX-970 | | 1.69 | 1.56 | 2.04 | 0.98 | 0.94 | 1.24 | 0.120 | 0.482 | 0.347 | 0.49 | 0.58 | 0.67 | 0.58 | 0.85 | 1.06 |
| 1276 | TMZ/VX-970 | | 1.52 | 2.49 | 2.40 | 1.07 | 1.52 | 1.60 | 0.533 | 0.938 | 0.245 | 0.53 | 0.50 | 0.76 | 0.78 | 0.64 | 0.93 |
| 1286 | TMZ/VX-970 | | 2.59 | 2.58 | 3.20 | 1.66 | 1.67 | 2.06 | 1.060 | 1.914 | 1.896 | 0.88 | 0.47 | 0.75 | 1.37 | 0.67 | 1.29 |
| 1292 | TMZ/VX-970 | | 2.41 | 2.36 | 3.89 | 1.64 | 1.56 | 2.28 | 0.981 | 1.027 | 0.717 | 0.66 | 0.77 | 1.74 | 0.84 | 0.98 | 2.43 |
| 1295 | TMZ/VX-970 | | 2.95 | 2.86 | 2.50 | 1.87 | 1.75 | 1.67 | 0.510 | 0.487 | 0.581 | 0.72 | 0.62 | 0.49 | 1.01 | 0.73 | 0.84 |

| **BL0479-F1894** | |  | |  | |  | |  | | |  | |  | |  |  |  |  |  |  |  |  |  |  |
| --- | --- | --- | --- | --- | --- | --- | --- | --- | --- | --- | --- | --- | --- | --- | --- | --- | --- | --- | --- | --- | --- | --- | --- | --- |
|  | |  | **SUVbw(max)** | | | | | | **SUVbw(mean-50%)** | | | | | **%ID** | | | | **Tumor/Liver** | | | **Tumor/Tissue** | | |  |
| **M#** | **Group** | | **Day 0** | | **Day 3** | | **Day 8** | | **Day 0** | **Day 3** | | **Day 8** | | **Day 0** | | **Day 3** | **Day 8** | **Day 0** | **Day 3** | **Day 8** | **Day 0** | **Day 3** | **Day 8** |  |
| 1057 | Vehicle | | 0.98 | | 1.43 | | 1.07 | | 0.68 | 1.00 | | 0.73 | | 0.041 | | 0.028 | 0.028 | 2.36 | 1.81 | 2.06 | 3.99 | 1.63 | 3.01 |  |
| 1065 | Vehicle | | 2.64 | | 2.70 | | 2.22 | | 1.72 | 1.71 | | 1.42 | | 0.232 | | 0.318 | 0.246 | 2.98 | 3.19 | 2.72 | 4.09 | 3.64 | 2.63 |  |
| 1068 | Vehicle | | 1.76 | | 2.52 | | 1.59 | | 1.14 | 1.63 | | 0.99 | | 0.140 | | 0.225 | 0.082 | 1.56 | 1.98 | 3.60 | 2.35 | 2.37 | 7.67 |  |
| 1070 | Vehicle | | 1.85 | | 2.18 | | 1.87 | | 1.23 | 1.38 | | 1.21 | | 0.102 | | 0.109 | 0.081 | 2.38 | 2.12 | 3.30 | 3.34 | 2.68 | 3.64 |  |
| 1077 | Vehicle | | 1.52 | | 3.51 | | 2.71 | | 1.02 | 2.32 | | 1.77 | | 0.091 | | 0.148 | 0.282 | 2.46 | 1.66 | 2.01 | 2.41 | 1.79 | 2.42 |  |
| 1080 | Vehicle | | 2.55 | | 3.34 | | 3.19 | | 1.63 | 2.09 | | 2.01 | | 0.612 | | 0.720 | 0.427 | 2.58 | 2.00 | 4.18 | 2.83 | 2.75 | 3.99 |  |
| 1059 | TMZ/VX-970 | | 2.59 | | 1.99 | | 2.00 | | 1.81 | 1.28 | | 1.31 | | 0.053 | | 0.089 | 0.044 | 1.50 | 1.15 | 1.60 | 1.87 | 1.31 | 1.80 |  |
| 1060 | TMZ/VX-970 | | 2.26 | | 2.53 | | 1.61 | | 1.46 | 1.59 | | 1.03 | | 0.159 | | 0.128 | 0.075 | 1.41 | 1.27 | 1.29 | 1.63 | 1.71 | 1.37 |  |
| 1069 | TMZ/VX-970 | | 2.75 | | 2.16 | | 1.63 | | 1.67 | 1.35 | | 1.04 | | 0.238 | | 0.294 | 0.112 | 1.41 | 1.31 | 2.65 | 1.96 | 1.58 | 4.50 |  |
| 1073 | TMZ/VX-970 | | 2.92 | | 3.45 | | 1.47 | | 1.98 | 2.42 | | 0.94 | | 0.435 | | 0.880 | 0.124 | 3.02 | 1.14 | 1.37 | 3.27 | 1.47 | 2.03 |  |
| 1075 | TMZ/VX-970 | | 1.95 | | 2.21 | | 2.09 | | 1.30 | 1.46 | | 1.31 | | 0.247 | | 0.401 | 0.108 | 2.62 | 1.82 | 1.84 | 3.48 | 2.12 | 1.94 |  |
| 1083 | TMZ/VX-970 | | 1.57 | | 2.44 | | 0.95 | | 1.05 | 1.58 | | 0.61 | | 0.131 | | 0.149 | 0.050 | 1.52 | 2.13 | 1.85 | 1.75 | 2.67 | 1.63 |  |

| **765638-272-R** | |  |  |  |  |  |  |  |  |  |  |  |  |  |  |  |  |
| --- | --- | --- | --- | --- | --- | --- | --- | --- | --- | --- | --- | --- | --- | --- | --- | --- | --- |
|  | |  | **SUVbw(max)** | | | **SUVbw(mean-50%)** | | | **%ID** | | | **Tumor/Liver** | | | **Tumor/Tissue** | | |
| **M#** | **Group** | | **Day 0** | **Day 3** | **Day 8** | **Day 0** | **Day 3** | **Day 8** | **Day 0** | **Day 3** | **Day 8** | **Day 0** | **Day 3** | **Day 8** | **Day 0** | **Day 3** | **Day 8** |
| 1043 | Vehicle | | 0.87 | 0.92 | 1.07 | 0.59 | 0.55 | 0.60 | 0.429 | 0.321 | 0.409 | 0.58 | 0.55 | 0.46 | 0.66 | 0.67 | 0.57 |
| 1046 | Vehicle | | 1.31 | 0.72 | 0.68 | 0.72 | 0.48 | 0.45 | 0.596 | 0.792 | 1.001 | 1.06 | 0.83 | 0.74 | 1.25 | 1.02 | 0.82 |
| 1047 | Vehicle | | 0.96 | 0.81 | 0.78 | 0.68 | 0.60 | 0.56 | 0.749 | 0.768 | 0.673 | 0.75 | 0.75 | 0.89 | 0.93 | 0.93 | 1.08 |
| 1052 | Vehicle | | 1.42 | 0.67 | 0.69 | 0.60 | 0.44 | 0.52 | 0.413 | 0.374 | 0.904 | 0.77 | 0.78 | 0.85 | 0.90 | 0.99 | 1.02 |
| 1055 | Vehicle | | 1.42 | 0.75 | 0.60 | 0.83 | 0.52 | 0.41 | 0.160 | 0.860 | 0.832 | 0.45 | 0.85 | 0.69 | 0.61 | 1.13 | 0.81 |
| 1036 | Cisplatin/VX-970 | | 1.12 | 1.66 | 2.16 | 0.79 | 1.09 | 1.29 | 0.581 | 0.707 | 0.565 | 0.54 | 0.32 | 0.49 | 0.75 | 0.52 | 0.62 |
| 1038 | Cisplatin/VX-970 | | 0.92 | 1.06 |  | 0.63 | 0.63 |  | 0.329 | 0.279 |  | 0.56 | 0.70 |  | 0.73 | 1.21 |  |
| 1040 | Cisplatin/VX-970 | | 0.78 |  |  | 0.51 |  |  | 0.527 |  |  | 0.58 |  |  | 0.70 |  |  |
| 1042 | Cisplatin/VX-970 | | 0.84 | 1.37 | 1.00 | 0.52 | 0.73 | 0.62 | 0.642 | 0.789 | 0.377 | 0.51 | 0.50 | 0.42 | 0.58 | 0.65 | 0.56 |
| 1049 | Cisplatin/VX-970 | | 1.46 | 1.05 | 0.83 | 0.79 | 0.69 | 0.50 | 0.266 | 0.660 | 0.448 | 0.86 | 0.78 | 0.66 | 1.25 | 1.04 | 0.80 |
| 1051 | Cisplatin/VX-970 | | 0.84 | 0.92 | 1.01 | 0.55 | 0.66 | 0.63 | 0.526 |  | 0.673 | 0.70 | 0.85 | 0.52 | 0.97 | 1.13 | 0.70 |
| 1054 | Cisplatin/VX-970 | | 1.16 |  | 0.96 | 0.71 |  | 0.64 | 0.911 |  |  | 0.73 |  | 0.58 | 1.10 |  | 0.75 |
